# Prediction of the potential distribution of *Chimonobambusautilis* (Poaceae, Bambusoideae) in China, based on the MaxEnt model

**DOI:** 10.3897/BDJ.12.e126620

**Published:** 2024-06-24

**Authors:** Guangneng Yang, Na Liu, Xu Zhang, Hua Zhou, Yiju Hou, Peng Wu, Xi Zhang

**Affiliations:** 1 Guizhou Provincial Academy of Forestry, Guiyang, China Guizhou Provincial Academy of Forestry Guiyang China; 2 Guizhou Caohai Observation and Research Station for Wet Ecosystem, National Forestry and Grassland Administration, Weining, China Guizhou Caohai Observation and Research Station for Wet Ecosystem, National Forestry and Grassland Administration Weining China; 3 Guizhou Liping Observation and Research Station for Karst Rocky Desert Ecosystem, Qiandongnan Prefecture, China Guizhou Liping Observation and Research Station for Karst Rocky Desert Ecosystem Qiandongnan Prefecture China; 4 Guizhou Libo Observation and Research Station for Karst Forest Ecosystem, Libo, China Guizhou Libo Observation and Research Station for Karst Forest Ecosystem Libo China; 5 Chishui Bamboo Forest Ecosystem National Observation and Research Station, Chishui, China Chishui Bamboo Forest Ecosystem National Observation and Research Station Chishui China

**Keywords:** suitable habitat, the maximum entropy model (MaxEnt), *Chimonobambusautilis*, bamboo distribution

## Abstract

*Chimonobambusautilis* is a unique edible bamboo species valued for its economic and nutritional benefits. However, its existence in natural habitats is at risk due to environmental shifts and human interventions. This research utilised the maximum entropy model (MaxEnt) to predict potential habitats for *Ch.utilis* in China, identifying key environmental factors influencing its distribution and analysing changes in suitable habitats under future climate conditions. The results show that the results of the MaxEnt model have high prediction accuracy, with an AUC (Area Under the receiver operating characteristic Curve) value of 0.997. Precipitation in the driest month (Bio14), altitude (Alt) and isothermality (Bio03) emerged as the primary environmental factors influencing the *Ch.utilis* distribution. Currently, the suitable habitats area for *Ch.utilis* is 10.55 × 10^4^ km^2^. Projections for the 2050s and 2090s indicate potential changes in suitable habitats ranging from -3.79% to 10.52%. In general, the most suitable habitat area will decrease and shrink towards higher latitude areas in the future. This study provides a scientific basis for the introduction, cultivation and conservation of *Ch.utilis*.

## Introduction

Vegetation is the basis of terrestrial ecosystems and its distribution is limited by climate (Çoban et al. 2020, Jian et al. 2022). Over the past century, climate change has triggered a series of environmental problems that have significantly impacted terrestrial ecosystems (Moritz and Agudo 2013, Li et al. 2019), such as global warming and changes in the geographical distribution of species (Farahat and Refaat 2021, Varol et al. 2021). The studies found that global warming will prompt species to move towards cooler regions, expanding to higher latitudes, altitudes and deeper ocean waters (Lawlor et al. 2024). It is projected that around 57% of widely distributed plant species will experience a reduction of more than 50% in their climatically suitable range by 2080 due to climate warming (Warren et al. 2013). Numerous studies have demonstrated that climate change has a profound impact on species distribution ranges (Parmesan and Yohe 2003, Subba et al. 2018, Oduor et al. 2023). Therefore, it is essential to research the distribution of vegetation under changing climatic conditions, identify migration patterns and develop strategies for the introduction, cultivation and conservation of species. This approach can help prevent resource waste and losses that may occur from uninformed introductions (Gui et al. 2024, Li et al. 2024, Wang et al. 2024).

*Chimonobambusautilis*, a member of the Poaceae bamboo subfamily, is a unique edible bamboo species found in south-western China (Geng and Wang 1996, Liu et al. 2022), mainly in Sichuan, Guizhou and Yunnan Provinces, as well as Chongqing Municipality, within an altitude range of 1400 m to 2200 m (Wu and Han 1982, Zhang et al. 2005). The bamboo shoot of *Ch.utilis* has high nutritional value, becoming a food in people’s daily lives (Li et al. 2008). Additionally, this bamboo serves as a significant garden plant in karst environments due to its ornamental value and it is a valuable raw material for paper-making and bamboo weaving. However, the wild *Ch.utilis* population is facing degradation as a result of environmental changes and human activities, failing to meet local economic demands. To solve this problem, *Ch.utilis* has been extensively cultivated in south-western China, playing a crucial role in economic and industrial development in the region (Liu et al. 2022). Current research on *Ch.utilis* has predominantly focused on forest management, growth and pest control (Zhang et al. 1997, Yi et al. 2009, Ding et al. 2011). While some studies have examined the suitable areas for *Ch.utilis* in Tongzi County under present climatic conditions, these smaller-scale studies have downplayed the significance of precipitation and temperature, leading to certain limitations (Mo and Luo 2005, Li et al. 2011). The impact of climate change on species distribution is a widely studied topic, yet the specific effects on *Ch.utilis* remain unclear. It is imperative to analyse the geographic distribution of *Ch.utilis* in China in light of climate change to formulate effective conservation strategies for wild populations and maximise their ecological and economic benefits.

With the advancement of scientific technology, species distribution models (SDMs) have gained popularity as valuable tools for studying the impact of climate change on species (Elith and Leathwick 2009, Booth 2018, Zheng et al. 2024). One of the most widely used SDMs is the maximum entropy model (MaxEnt) (Liu et al. 2021), which evaluates habitat suitability, based on species distribution coordinates and environmental data (Dai et al. 2022). Being a machine-learning algorithm, the model provides accurate predictions and ease of use (Pan et al. 2020, Jian et al. 2022), frequently used for predicting species distributions, protecting rare plants and animals and managing invasive species spread (Wang et al. 2021, Ma et al. 2022, Zhang et al. 2022, Hosni et al. 2024). By combining the MaxEnt model with ArcGIS, researchers can analyse potential species distribution changes due to climate change, offering valuable insights for developing effective strategies to mitigate its impact on species by scientists and policy-makers.

The study hypothesises that climate change will have a significant impact on the distribution of *Ch.utilis*. Based on the species' current distribution points and environmental variables from global climate models and utilising the MaxEnt model and ArcGIS 10.5, the research aims to address the following questions: (1) What are the limiting factors and distribution ranges for *Ch.utilis* and (2) How will suitable habitats change in the future (2050s and 2090s)? This study could provide a theoretical foundation and practical guidance for the introduction, cultivation and conservation of *Ch.utilis*.

## Materials and methods

### Acquisition of species distribution data

**Figure 1.**
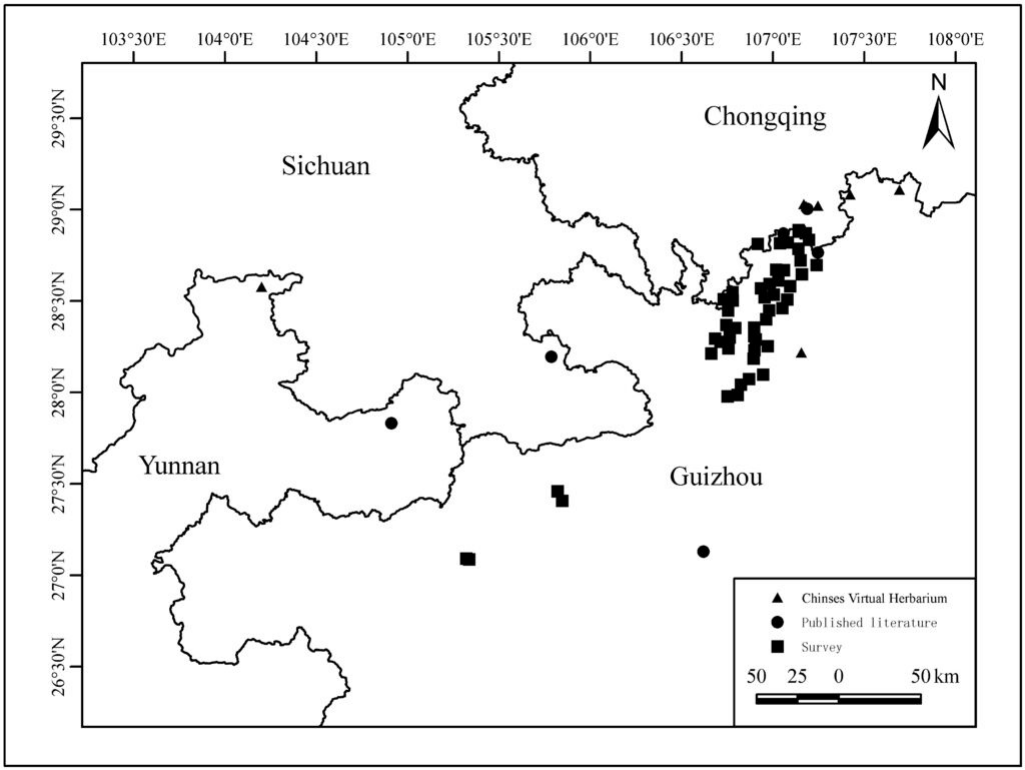
Distribution of *Ch.utilis* in China.

China was selected as the research area to analyse the distribution of *Ch.utilis*. Field investigations were carried out from 2020 to 2023 to study the natural population of *Ch.utilis*, resulting in the collection of 582 distribution records. Additionally, a review of published literature retrieved 19 records on the natural population distribution of *Ch.utilis* in Yunnan and Guizhou Provinces (Zhang et al. 2019, Lou 2021b, Wang et al. 2022). The Chinese Virtual Herbarium (CVH, www.cvh.ac.cn/, accessed on 19 September 2022) provided 121 records. *Ch.utilis* was introduced and cultivated in 2000 as part of the farmland-to-forest project. To mitigate the impact of this project, records from 2000 onwards sourced from the CVH were excluded. Following the removal of duplicate records, only one distribution point was retained for each grid (1 km × 1 km), resulting in 62 valid samples (Fig. 1).

### Acquisition of environmental data

In this study, a total of 19 climate variables, three terrain variables and four soil variables were utilised to develop the MaxEnt model (Table 1). The climate data were sourced from the World Climate Database (WorldClim v.2.1, www.worldclim.org/, accessed on 22 September 2022), encompassing current (1970s - 2000s), future 2050s (2040s - 2060s) and future 2090s (2080s - 2100s) scenarios, with a spatial resolution of 30″, approximately 1 km × 1 km. Future climate data were based on the MIROC6 (Model for Interdisciplinary Research On Climate version 6) model in CMIP6 (Coupled Model Intercomparison Project Phase 6), incorporating SSP1_2.6 (SSP1, sustainable development path), SSP2_4.5 (SSP2, medium development path) and SSP5_8.5 (SSP5, conventional development path) scenarios (Riahi et al. 2017, Zhang et al. 2022, Li et al. 2022). The global elevation model (DEM) data, with a resolution of approximately 1 km × 1 km, was also obtained from the World Climate Database, while altitude, aspect and slope data were derived from the DEM. Furthermore, four soil variables were extracted from the Harmonised World Soil Database (www.fao.org/soils-portal/soil-survey/soil-maps-and-databases/harmonized-world-soil-database-v12/en/, accessed on 25 March 2024). It was assumed that the terrain and soil variables remained constant when predicting future suitable habitats.

In order to prevent overfitting, a comprehensive assessment of 26 climate variables was conducted. The screening process consisted of two main steps: (1) Utilising SPSS 23.0 software to examine the correlation amongst the 26 climate variables, with a threshold of 0.80 set for determination; and (2) Running MaxEnt 3.4.1 software with species distribution data and the 26 climate variables to determine the initial percentage contribution of each variable to the model. Following this, variables with a correlation coefficient above 0.80 and a lower contribution rate were excluded (Fang et al. 2022). Ultimately, eleven climate variable factors were selected for the subsequent prediction of Ch.utilis distribution (Table 1).

### MaxEnt modelling

The prediction of the potential distribution area of *Ch.utilis* was conducted using the MaxEnt model. The MaxEnt model derived constraint conditions based on the distribution data of species and environmental factors. It assumes that the probability distribution of species emergence is closest to its actual distribution when the entropy is maximised under these constraints (Phillips et al. 2006). In this study, distribution data for 62 *Ch.utilis* and 26 environmental variables were input into MaxEnt 3.4.1 software. A total of 25% of the distribution points were utilised as test data, while 75% were used as training data. The simulation was repeated 10 times with other parameters remaining constant.

### Model accuracy evaluation

The prediction accuracy was evaluated using the receiver operating characteristic (ROC) curve and the area under the ROC (AUC). AUC values range from 0 to 1, with higher values indicating better prediction accuracy. Specific criteria were defined as follows: 0.5-0.6 for failure, 0.6-0.7 for poor, 0.7-0.8 for fair, 0.8-0.9 for good and 0.9-1 for excellent. (Araújo et al. 2005, Pan et al. 2020).

### Classification of potentially suitable areas

The output results of the MaxEnt model were visualised using ArcGIS 10.5 software to analyse habitat suitability. Habitat suitability was assessed by categorising habitat into four levels using the natural-breaks classification method: unsuitable habitat (0-0.05), marginally suitable habitat (0.05-0.22), highly suitable habitat (0.22-0.51) and most suitable habitat (0.51-1). The suitable habitat for each province was determined by overlaying administrative divisions with suitable areas. Finally, a transfer matrix was utilised to analyse the relationship between the current suitable habitat and the future suitable habitat.

## Results

### Model accuracy evaluation

**Figure 2.**
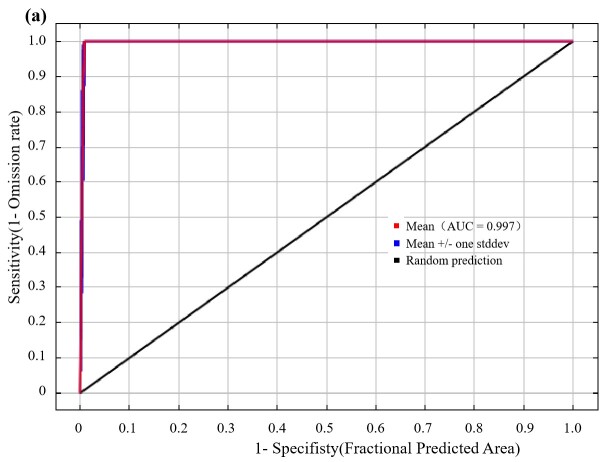
Receiver operating characteristic curve of *Ch.utilis* for MaxEnt model.

The MaxEnt model was utilised to predict potentially suitable habitats for *Ch.utilis*. The evaluation of the ROC curve results showed that the average AUC value of the training data was 0.997, indicating high reliability in the prediction outcomes. (Fig. 2).

### Dominant environmental factors

The precipitation in the driest month (Bio14) made the highest percentage contribution to the prediction model at 45.70%. This was followed by altitude (Alt) and isothermality (Bio03), which contributed 33.60% and 17.70%, respectively, resulting in a cumulative contribution of 97.00%. The contributions of the other factors were minimal (Table 2).

### Environmental characteristics of Ch.utilis

**Figure 3.**
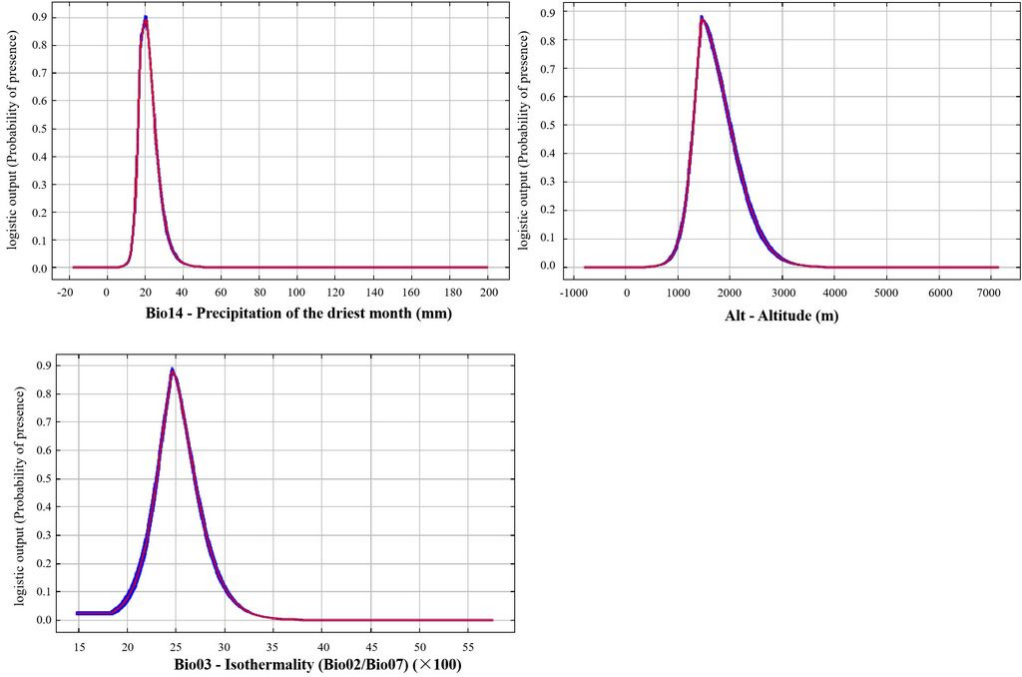
Response curves of the probability of the main climate factors.

The curve of the distribution probability and environmental factor response, as shown in Fig. 3, indicates a pattern of slow increase, rapid increase, rapid decrease and slow decrease with increasing environmental factor values. The probability of occurrence exceeded 0.50. The environmental characteristics within the distribution area of *Ch.utilis* included a range of precipitation in the driest month (Bio14) of 16.31-25.26 mm, an altitude (Alt) range of 1288.86-2005.11 m and an isothermality (Bio03) range of 23.03-26.91.

### Distribution of suitable habitats for Ch.utilis in China under the current climate

**Figure 4.**
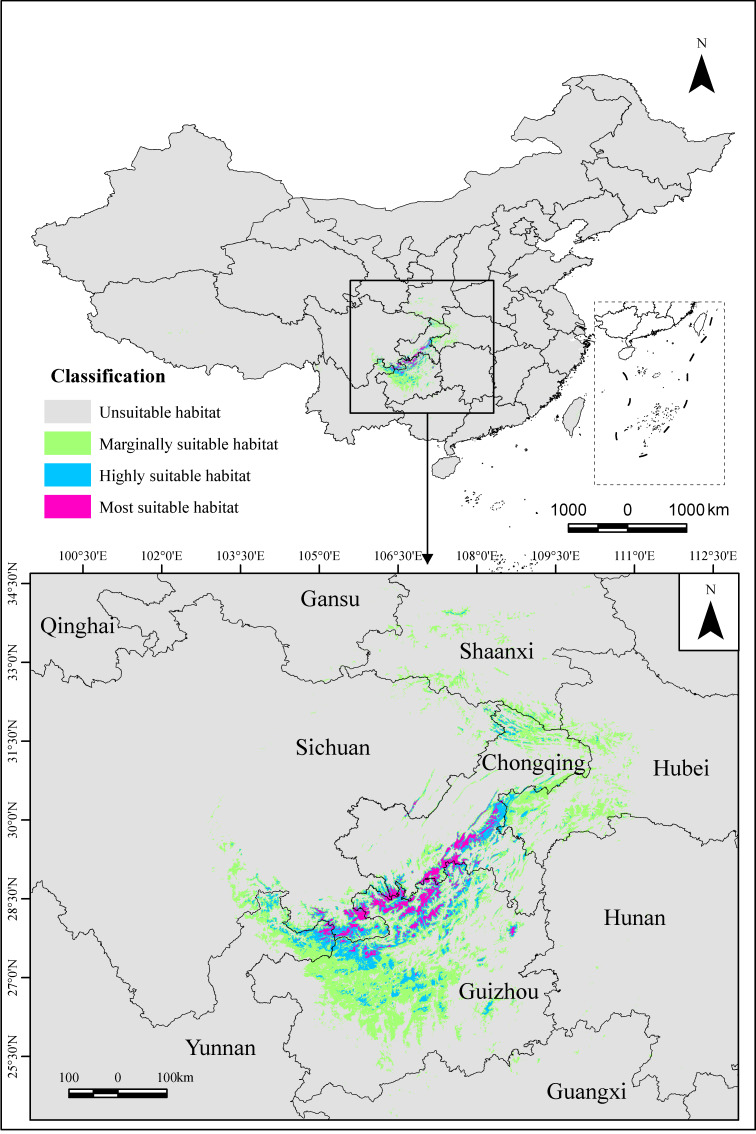
Potential distribution of *Ch.utilis* in China under the current climate.

The total suitable area of *Ch.utilis* in China under the current climate was 10.55 × 10^4^ km^2^ (Table 3 and Fig. 4). Suitable habitats were predominantly found in the south-western region of China, encompassing north-eastern Yunnan Province, south-eastern Sichuan Province, most of Guizhou Province, southern and northern Chongqing Municipality, western Hubei Province and southern Shanxi Province.

The most suitable habitat area was 0.85 × 10^4^ km^2^, accounting for 8.06% of the total suitable area. These habitats were primarily located in the junctional areas of Yunnan, Guizhou, Sichuan Provinces and Chongqing Municipality, specifically in the Daloushan Mountains and Wumeng Mountains. The highly suitable habitat area was 2.16 × 10^4^ km^2^, making up 20.47% of the total suitable area. These habitats were mainly found in north-eastern Yunnan Provinces, south-eastern Sichuan Provinces, the central and northern parts of Guizhou Provinces, and the southern and northern parts of Chongqing Municipality. The marginally suitable habitat area was 7.54 × 10^4^ km^2^, accounting for 71.47% of the total suitable area. It is predominantly found in south-western Hubei Province, southern Shaanxi Province, north-eastern Chongqing Municipality, north-eastern Yunnan Province, south-eastern Sichuan Province and most of Guizhou Province. A comparison with the natural distribution revealed that the model predicted a much larger range. Despite some deviations, the core distribution area matched the current distribution area.

### Future changes in habitat suitability

We predicted the potential distribution of *Ch.utilis* in China in two future periods (2050s and 2090s) and three greenhouse gas scenarios (SSP1, SSP2 and SSP5).

**Figure 5.**
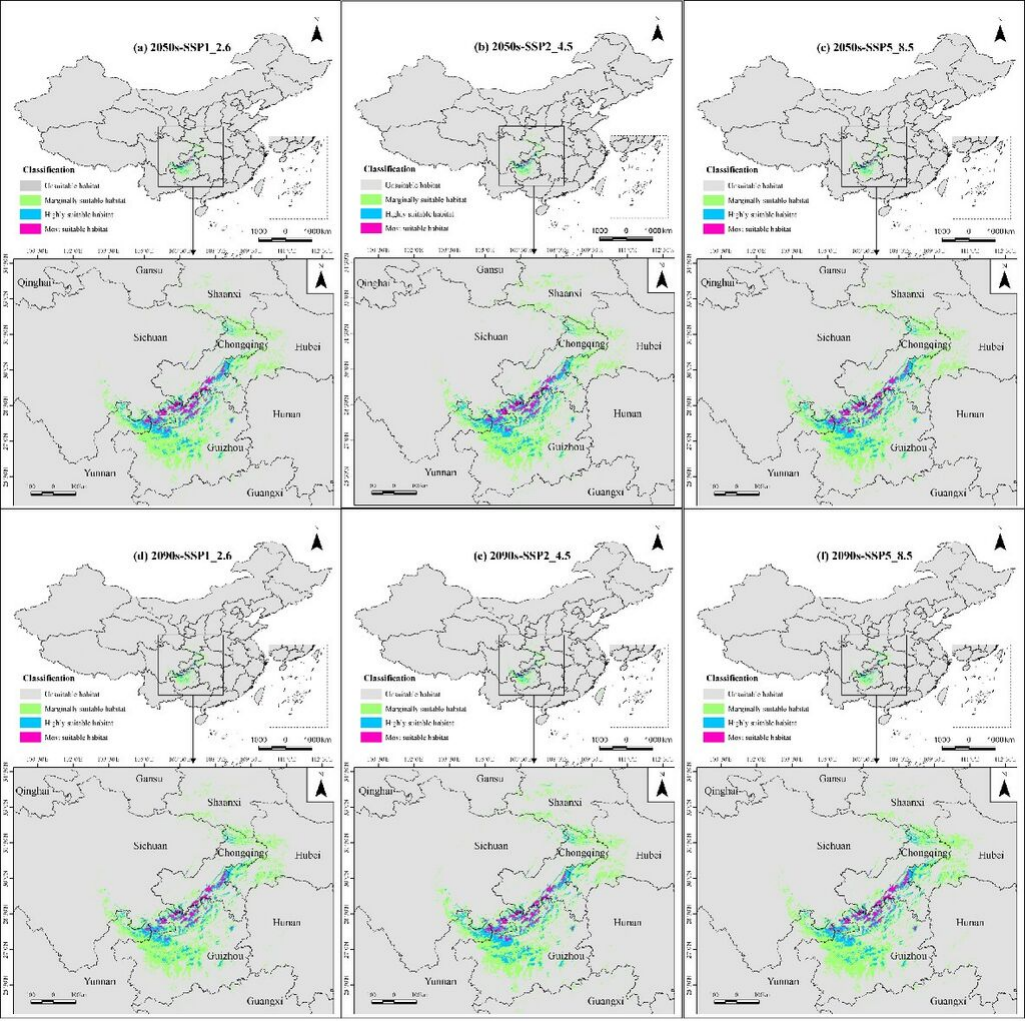
Potential distribution of *Ch.utilis* under future climate scenarios. Panels (a, b, c) indicate the potential distributions under the three scenarios in the 2050s; panels (d, e, f) indicate the potential distributions under the three scenarios in the 2090s.

In the 2050s and 2090s, the total suitable habitat area for *Ch.utilis* decreased by 3.79% and 1.04%, respectively, under the SSP1 scenario, reaching 10.15 × 10^4^ km^2^ and 10.44 × 10^4^ km^2,^ respectively (Table 3 and Fig. 5). Conversely, the total area increased by 0.38% and 6.16% under the SSP2 scenario, totalling 10.59 × 10^4^ km^2^ and 11.20 × 10^4^ km^2^, respectively, for the same periods. Similarly, under the SSP5 scenario, the total area expanded by 1.14% and 10.52%, reaching 10.67 × 10^4^ km^2^ and 11.66 × 10^4^ km^2^ for the 2050s and 2090s, respectively. The most suitable habitats for *Ch.utilis* generally decreased, except in the 2050s-SSP1 scenario. While the highly suitable habitats decreased under the SSP1 scenario, they increased under the SSP2 and SSP5 scenarios. Conversely, marginally suitable habitat areas increased, except in the 2050s-SSP1 scenario. Generally, in the future, the suitable habitat area will decrease in the southern region (including Yunnan, Guizhou, Hunan and Hubei Provinces; Chongqing Municipality; and Guangxi Zhuang Autonomous Region), but increase in the northern region (such as Gansu, Sichuan and Shaanxi Provinces).

To highlight the changes in suitable area between the current and future scenarios, we used a transition matrix to analyse internal changes (in the current and 2090s) without considering the total area of unsuitable areas.

Under the SSP1 scenario, the area of grade change from the present to the 2090s was 11.27 × 10^4^ km^2^ (Table 4). Specifically, the marginally suitable habitat of 1.04 × 10^4^ km^2^ transitioned into unsuitable habitat (0.83 × 10^4^ km^2^) and highly suitable habitat (0.21 × 10^4^ km^2^). The highly suitable habitat area of 0.57 × 10^4^ km^2^ transformed into marginal habitat (0.50 × 10^4^ km^2^) and most suitable habitat (0.07 × 10^4^ km^2^), while the most suitable habitat area of 0.20 × 10^4^ km^2^ transitioned into highly suitable habitat. Additionally, an area of 1.00 × 10^4^ km^2^ became a more suitable habitat, with 0.72 × 10^4^ km^2^ of this area becoming a new suitable habitat. Conversely, the habitat quality decreased by an area of 1.53 × 10^4^ km^2^, with 0.83 × 10^4^ km^2^ of suitable habitat being lost.

Under the SSP2 scenario, the area of grade change from the present to the 2090s was 11.81 × 10^4^ km^2^ (Table 5). The marginally suitable habitat with an area of 1.14 × 10^4^ km^2^ became unsuitable habitat (0.61 × 10^4^ km^2^) and highly suitable habitat (0.53 × 10^4^ km^2^), the highly suitable habitat with an area of 0.46 × 10^4^ km^2^ became marginally suitable habitat (0.34 × 10^4^ km^2^) and most suitable habitat (0.12 × 10^4^ km^2^) and the most suitable habitat with an area of 0.14 × 10^4^ km^2^ became the highly suitable habitat. An area of 1.91 × 10^4^ km^2^ became a more suitable habitat, of which 1.26 × 10^4^ km^2^ of the area became a new suitable habitat. Conversely, the habitat quality decreased by 1.09 × 10^4^ km^2^ and 0.61 × 10^4^ km^2^ of suitable habitat was lost.

Under the SSP5 scenario, the area of grade change from the present to the 2090s was 12.17 × 10^4^ km^2^ (Table 6). The marginally suitable habitat with an area of 0.89 × 10^4^ km^2^ became unsuitable habitat (0.51 × 10^4^ km^2^), the highly suitable habitat (0.38 × 10^4^ km^2^), the highly suitable habitat with an area of 0.39 × 10^4^ km^2^ became marginally suitable habitat (0.30 × 10^4^ km^2^) and the most suitable habitat (0.09 × 10^4^ km^2^) and the most suitable habitat with an area of 0.15 × 10^4^ km^2^ became the highly suitable habitat. An area of 2.09 × 10^4^ km^2^ became a more suitable habitat, of which 1.62 × 10^4^ km^2^ became a new suitable habitat. Conversely, the habitat quality decreased by 1.26 × 10^4^ km^2^ and 0.51 × 10^4^ km^2^ of suitable habitat was lost.

## Discussion

### Environmental factors affecting the distribution of Ch.utilis

Amongst the 11 environmental factors considered in the modelling process, precipitation had the highest total contribution rate at 46.00%, followed by terrain at 33.70%, temperature at 19.10% and soil at 1.20% (Table 2). This indicates that precipitation and terrain were the primary factors influencing the distribution of *Ch.utilis*. The results from the Jackknife test and single response curves revealed that the precipitation of the driest month (Bio14) was the most significant factor, with an optimal growth range between 16.31-25.26 mm, followed by altitude (Alt, 1288.86-2005.11 m) and isothermality (Bio03, 23.03-26.91) (Fig. 3). Previous studies have indicated that precipitation is a crucial environmental factor influencing the distribution and growth of bamboo (Zhou 1991), which is in line with the findings of this study (Lou 1991, Zhou 1991, Li et al. 2019). Specifically, precipitation during the driest month emerged as the primary environmental factor shaping the distribution of *Ch.utilis*, showing a high contribution rate and significant permutation importance in the MaxEnt model. It has the same habitat preference as *Chimonobambusahejiangensis* (Wang et al. 2019). Furthermore, because of its strong adaptability to soil, the contribution rate related to soil was small (Liu et al. 2022). Currently, the natural *Ch.utilis* is mainly naturally distributed in south-western China within an altitude range of 1400 m to 2200 m (Ding et al. 2011). The model predicts that the optimal growth range altitude for the bamboo species is between 1288.86-2005.11 m, suggesting that it may be found in low-altitude areas. The differences in distribution at high altitudes may be attributed to the limited sample size used in model construction (62) and the incomplete coverage of the species' entire environmental range in the sample data, impacting prediction results (Chen et al. 2012). As the sample size increases, prediction accuracy follows a non-linear pattern and eventually stabilises (Ji et al. 2019). To improve predictive accuracy, it is advised to incorporate training data that spans the species' entire environmental range when utilising the MaxEnt model for habitat predictions.

### Suitable habitats for Ch.utilis under the current climate

Under the current climate, the total suitable habitat area for *Ch.utilis* in China is 10.55×10^4^ km^2^ (Table 3), with the most suitable habitats mainly concentrated in the junction area of Guizhou, Sichuan Province and Chongqing Municipality (Fig. 4). These regions are located in the transition area between the Sichuan Basin and the Yunnan-Guizhou Plateau, characterised by a sub-tropical monsoon climate with hot and rainy summers and mild and humid winters. This climate aligns with *Ch.utilis*'s preference for low temperatures and high precipitation (Wu and Han 1982, Lou 1991). Research indicates that the most suitable habitats are likely to harbour rich genetic diversity and are core regions for germplasm resource distribution (Wu et al. 2018). Therefore, it is recommended to prioritise investigations, resource collection and protection efforts in these regions. The highly suitable habitat for *Ch.utilis* is mainly found in north-eastern Yunnan Guizhou, south-eastern Sichuan Guizhou, most of Guizhou Province and the southern and northern parts of Chongqing Municipality. These regions provide suitable conditions for the large-scale introduction and domestication of *Ch.utilis* (such as Dafang County and Qingzhen City in Guizhou Province and Chaotian and Zhaohua areas of Guangyuan City at the junction of Sichuan, Shaanxi and Gansu Provinces) (Lou 2021a). Moreover, it is recommended to conduct resource surveys in these areas to investigate the possible existence of wild *Ch.utilis* populations. There were also many areas of marginally suitable habitats in western Hubei Province, south-western Shaanxi Province, south-eastern Sichuan Province, south-western Chongqing Municipality, most parts of Guizhou Province and south-western Taiwan Province, where the actual local conditions should be considered for the introduction of *Ch.utilis*.

### Changes in the suitable habitats of Ch.utilis under future climate scenarios

From the current to the 2050s, the area of *Ch.utilis* varied from -3.79% to 1.14%, with a noticeable expansion only observed under the SSP5 scenario. Moving forward to the 2090s, the suitable habitat area for *Ch.utilis* varied from -1.04% to 10.52%, showing an expansion trend under both the SSP2 and SSP5 scenarios (Table 3). In the face of future climate change, the boundaries of *Ch.utilis*' suitable habitat underwent irregular changes, with a noticeable northward expansion trend, although the overall area change remained minimal. The suitable habitats in Yunnan, Hubei and Guizhou Provinces, Chongqing Municipality and Guangxi Zhuang Autonomous Region were significantly impacted by climate change in future scenarios, leading to a decrease. These changes are attributed to rising temperatures and falling precipitation in south-western China（Pan et al. 2020, Zhang et al. 2020）. Measures should be implemented to rescue and protect the existing *Ch.utilis* resources in these areas to prevent the loss of valuable germplasm resources. Additionally, it is essential to make efforts to introduce, cultivate and promote the existence of *Ch.utilis* in the above regions. Numerous studies have indicated a tendency for species' suitable habitats to shift towards higher latitudes and decrease in size due to climate change (Li et al. 2021, Li et al. 2022). In contrast, our research findings suggest a rising trend in the area of suitable bamboo habitats (Gui et al. 2024). Despite the emergence of new suitable habitats, the overall quality of the habitats is declining (Tables 4, 5, 6), potentially leading to a decrease or even disappearance of the most suitable habitat area if current trends persist (Rinawati et al. 2013, Jian et al. 2022).

## Conclusions

Our study revealed that the optimal habitats for *Ch.utilis* are primarily situated at the junctional regions of Yunnan, Guizhou, Sichuan Provinces and Chongqing Municipality, specifically within the Daloushan Mountains and Wumeng Mountains under both current and future climatic scenarios. The distribution of *Ch.utilis* is mainly influenced by precipitation in the driest month (Bio14), altitude (Alt) and isothermality (Bio03). While the total number of suitable habitats for *Ch.utilis* may slightly increase in the future, the overall range is not expected to change significantly. However, the most suitable habitat area is anticipated to decrease in the future. We recommend utilising the most suitable habitats as breeding grounds for germplasm resources, enhancing the investigation of germplasm resources and promoting large-scale artificial cultivation in highly suitable habitats. Additionally, conducting introduction experiments in marginally suitable habitats could provide valuable insights into how *Ch.utilis* responds to climate change.

## Figures and Tables

**Table 1.** Environmental factors.

Factor code	Description	Unit or description
*Alt*	*Altitude*	*m*
*Asp*	*Aspect*	-
*Slp*	*Slope*	°
Bio01	Annual mean temperature	°C
Bio02	Mean diurnal range (mean of monthly (max temp - min temp))	°C
*Bio03*	*Isothermality (Bio02/Bio07) (×100)*	*ratio*
Bio04	Temperature seasonality (standard deviation ×100)	standard deviation
Bio05	Max temperature of warmest month	°C
Bio06	Min temperature of coldest month	°C
*Bio07*	*Temperature annual range (Bio05-Bio06)*	°*C*
Bio08	Mean temperature of wettest quarter	°C
Bio09	Mean temperature of driest quarter	°C
Bio10	Mean temperature of warmest quarter	°C
Bio11	Mean temperature of coldest quarter	°C
Bio12	Annual precipitation	mm
*Bio13*	*Precipitation of wettest month*	*mm*
*Bio14*	*Precipitation of driest month*	*mm*
Bio15	Precipitation seasonality (coefficient of variation)	standard deviation
Bio16	Precipitation of wettest quarter	mm
Bio17	Precipitation of driest quarter	mm
Bio18	Precipitation of warmest quarter	mm
Bio19	Precipitation of coldest quarter	mm
*AWC*	*AWC range*	*mm/m*
*T_oc*	*Topsoil organic carbon*	%
*T_ph*	*Topsoil pH (H_2_O)*	-*log(H ^+^)*
*T_texture*	*Topsoil texture*	-

Note: * Italics text indicates the bioclimatic variables used for model construction.

**Table 2.** The percent contribution values of environmental variables.

Code	Percent contribution/%	Code	Percent contribution/%
Bio14	45.70	Bio13	0.30
Alt	33.60	Asp	0.10
Bio03	17.70	T_texture	0.00
Bio07	1.40	Slp	0.00
AWC	0.80	T_ph	0.00
T_oc	0.40		

**Table 3.** Prediction of suitable areas for *Ch.utilis* under future climate scenarios.

Scenarios	Marginally Suitable	Highly Suitable	Most Suitable	Total Suitable
	(10^4^ km^2^)	(10^4^ km^2^)	(10^4^ km^2^)	(10^4^ km^2^)
Current	7.54	2.16	0.85	10.55
2050s-SSP1	7.22	2.06	0.87	10.15
2050s-SSP2	7.63	2.16	0.80	10.59
2050s-SSP5	7.62	2.22	0.83	10.67
2090s-SSP1	7.72	2.00	0.72	10.44
2090s-SSP2	7.99	2.38	0.83	11.20
2090s-SSP5	8.57	2.30	0.79	11.66

**Table 4.** Transfer matrix of the suitable habitat area (current and 2090s-SSP1 scenario).

Period	2090s-SSP1					
	Grade	Unsuitable	Marginally suitable	Highly suitable	Most suitable	Total transferred
		(10^4^ km^2^)	(10^4^ km^2^)	(10^4^ km^2^)	(10^4^ km^2^)	(10^4^ km^2^)
Current	Unsuitable (10^4^ km^2^)	-	0.72	0.00	0.00	0.72
	Marginally suitable (10^4^ km^2^)	0.83	6.50	0.21	0.00	7.54
	Highly suitable (10^4^ km^2^)	0.00	0.50	1.59	0.07	2.16
	Most suitable (10^4^ km^2^)	0.00	0.00	0.20	0.65	0.85
	Total transferred	0.83	7.72	2.00	0.72	11.27

**Table 5.** Transfer matrix of suitable habitat area (current and 2090s-SSP2 scenario).

Period	2090s-SSP2					
	Grade	Unsuitable	Marginally Suitable	Highly Suitable	Most suitable	Total transferred
		(10^4^ km^2^)	(10^4^ km^2^)	(10^4^ km^2^)	(10^4^ km^2^)	(10^4^ km^2^)
Current	Unsuitable (10^4^ km^2^)	-	1.25	0.01	0.00	1.26
	Marginally Suitable (10^4^ km^2^)	0.61	6.40	0.53	0.00	7.54
	Highly Suitable (10^4^ km^2^)	0.00	0.34	1.70	0.12	2.16
	Most Suitable (10^4^ km^2^)	0.00	0.00	0.14	0.71	0.85
	Total transferred	0.61	7.99	2.38	0.83	11.81

**Table 6.** Transfer matrix of suitable habitat area (current and 2090s-SSP5 scenario).

Period	2090s-SSP5					
	Grade	Unsuitable Suitable	Marginally Suitable	Highly Suitable	Most suitable	Total transferred
		(10^4^ km^2^)	(10^4^ km^2^)	(10^4^ km^2^)	(10^4^ km^2^)	(10^4^ km^2^)
Current	Unsuitable (10^4^ km^2^)	-	1.62	0.00	0.00	1.62
	Marginally Suitable (10^4^ km^2^)	0.51	6.65	0.38	0.00	7.54
	Highly Suitable (10^4^ km^2^)	0.00	0.30	1.77	0.09	2.16
	Most Suitable (10^4^ km^2^)	0.00	0.00	0.15	0.70	0.85
	Total transferred	0.51	8.57	2.30	0.79	12.17
